# Corporate Co-Agglomeration and Green Economy Efficiency in China

**DOI:** 10.3389/fpsyg.2022.890214

**Published:** 2022-07-08

**Authors:** Xiaoyan Zhu, Yunqi Zhang, Weizhi Yang

**Affiliations:** School of Economics and Management, Sichuan Normal University, Chengdu, China

**Keywords:** corporate, co-agglomeration, green economy efficiency, manufacturing, producer services

## Abstract

This paper uses panel OLS, IV, and system GMM methods to empirically study the effects of manufacturing and producer service corporate co-agglomeration on green economy efficiency (GEE) in China. Chinese panel data from 2000 to 2019 are collected to assess the GEE and co-agglomeration degrees. The regression results show that there is an “inverted U-shaped” relationship between co-agglomeration and GEE. However, regional heterogeneity is found in the effects of corporate co-agglomeration on GEE. The mediating analysis indicates that corporate co-agglomeration could increase GEE through business entrepreneurship and innovation entrepreneurship. Variables such as transportation infrastructure, human capital, foreign direct investment, and environmental regulations are also found to have an elevating effect on GEE, whereas local fiscal expenditure on environmental protection has little effect. The findings in this paper indicate that entrepreneurship plays an important role in the process of co-agglomeration impacting GEE which differs in different regions and thus provide references for corporate and regional sustainable development.

## Introduction

Agglomeration refers to the corporate geographical proximity, that is, the co-location of corporations (Marshall, 1916). Manufacturing enterprises that produce the same products and upstream and downstream enterprises that produce related products tend to be geographically co-located, which is known as co-agglomeration (Ellison and Glaeser, 1997). Studies have shown that the location of a producer services sector is a function of the manufacturing sector location, that is manufacturing and associated producer service corporations are more likely to be closely located because producer services is an important input sector in manufacturing (Andersson, 2006). Producer services are intermediate industries that can be used in the further production of goods and services (Greenfield, 1966). Unlike the traditional service industries, which are consumer-oriented, producer services are producer-oriented. It includes finance, insurance, business services, and other knowledge-intensive industries (Browning and Singelmann, 1975). Producer services can directly or indirectly provide intermediate services to the manufacturing production process and link the various stages of production.

Therefore, manufacturing and producer service corporate co-agglomeration can enhance the core competitiveness of the manufacturing industries, cultivate a modern industrial organization system, and achieve a high level of development (Wang et al., 2022a). Accordingly, the co-agglomeration in this paper refers to the corporate co-agglomeration of producer services and manufacturing.

Co-agglomeration generates externalities, such as lower production costs (Fan and Scott, 2003), improved infrastructure, the promotion of competition, knowledge spillovers, and technological innovation (Marshall, 1916; Jacobs, 1969; Krugman, 1991; Porter, 1998). Co-agglomeration, through the above-mentioned externalities, can lead to higher productivity, which can drive economic growth (Lanaspa et al., 2016; Klein and Crafts, 2020), enhance the emissions reduction effects, and reduce environmental pollution (Zhuang et al., 2021; Wang et al., 2022b).

However, the co-location of many firms in a region can also lead to problems, such as traffic congestion, rising production factor prices, and insufficient market carrying capacities (Henderson, 1986; Brakman et al., 1996), all of which might inhibit productivity (Paci and Usai, 2000; Sbergami, 2002). Co-agglomeration can also result in high resource consumption and emissions, which could pollute the environment (Pandey and Seto, 2015; Lu et al., 2021).

Entrepreneurship, one of the externalities of co-agglomeration (Kemper et al., 2011; Wu et al., 2021), can also influence economic growth and the ecological environment (Glaeser and Kerr, 2009). Entrepreneurs can make better use of social capital, build corporate social relationships and mobilize employees, improve corporate productivity, and drive economic growth (Dastourian et al., 2017; Lafuente et al., 2020). Entrepreneurs also encourage firms to take greater social responsibility (Ahmad et al., 2021; Wei et al., 2021). Consequently, when regulated by social responsibility, companies will seek to achieve advances in clean energy technologies, which in turn can reduce environmental pollution (Zeng and Zhao, 2009; Otsuka et al., 2014).

Overall, co-agglomeration has been found to be highly correlated with economic growth and ecological environment (Rosenthal and Strange, 2020); however, in China, the effects of co-agglomeration on the two are not clear and widely debated (Lin and Tan, 2019). Therefore, this paper uses green economy efficiency (GEE) to represent the economic and environmental outputs (Qian and Liu, 2013; Naseer et al., 2021) to empirically verify the relationship between co-agglomeration and GEE in China and whether entrepreneurship plays a role in it.

This paper makes two main contributions. First, in the process of co-agglomeration affecting GEE, the role of entrepreneurship is studied. We verify that co-agglomeration enhances entrepreneurship, which boosts GEE by deploying social capital and assuming social responsibility. This provides a supplement to the study of regional green economy development. Second, geographic heterogeneity is taken into consideration in this paper. We find that the effects of co-agglomeration on GEE differ in different regions, which provides practical implications for regions to adjust the level of co-agglomeration and enhance GEE according to local conditions.

The remainder of this paper is organized as follows. Section Literature Review reviews the relevant literature on the impacts of manufacturing and producer service co-agglomeration on GEE, Section Empirical Analysis details the empirical study and variables, data and models are introduced, Section Empirical Results discusses the empirical results, assesses the regional heterogeneity, and analyzes the effects and mechanisms of co-agglomeration on GEE, and Section Conclusion gives the main conclusions.

## Literature Review

### Corporate Co-Agglomeration

Agglomeration, which is closely related to the development and evolution of industrial organizations, has been an important characteristic of economic development (Ellison et al., 2010; Chen and Chen, 2014). The essence of corporate co-agglomeration is the coordination of spatial locations and a geographic and spatial dimensional “synergetic clustering” (Jacobs et al., 2013; Gaubert, 2018). The co-agglomeration of corporates in manufacturing and producer services compresses the spatial industrial integration distances and allows for more interactive relationships between the two through mutual interaction and integration (Mansour and Kanso, 2017; Taghizadeh-Hesary et al., 2020).

Even though they have strong mutual needs, manufacturing and producer services can gradually become divided (Marshall, 1982). However, because producer services provide intermediate input factors, they have been increasingly integrated into manufacturing industries and all aspects of the manufacturing production chain (Lanaspa et al., 2016; Yang et al., 2018). This manufacturing and producer service co-agglomeration engenders closer cooperation and the product production process smoother. The industrial organization structure is also improved with the co-agglomeration of the two (Ganvir and Jain, 2021). Therefore, the corporate co-agglomeration between industries is a good way to build a new system of modern industrial organization, which benefits both economy and environment.

### Green Economy Efficiency

Whereas, rapid economic growth has led to increased regional spatial agglomeration, it has also resulted in more severe urban environmental problems (Zhang and Qin, 2018). Therefore, to establish a better ecological civilization and ensure a harmonious coexistence between people and nature, economic development needs to shift to a growth pattern that integrates economic growth, environmental protection, and resource conservation, that is, the “green economy” (Lin and Tan, 2019). The term “green economy” was first proposed by Pearce (1996) and is defined as an “affordable economy” in which the pursuit of economic growth does not lead to ecological crises.

The green economy is an economy that considers both the needs of society and the needs of the environment. However, a suitable index is required to effectively study the “green economy.” Many methods have been suggested to express the green economy, with the GEE being one of the most popular, and have been widely used, as it considers both the desirable and undesirable production outputs (Sahoo and Tone, 2008; Mendelová, 2022) and comprehensively considers both the resource and environmental costs. Therefore, the GEE is chosen to assess the state of the green economy.

### Co-Agglomeration and Green Economy Efficiency

The impact of co-agglomeration on GEE has been widely examined, but its exact effects remain controversial. There are three types of opinions on the co-agglomeration GEE relationship.

Some studies have found that manufacturing and producer service co-agglomeration can improve GEE (Ehrenfeld, 2003) in four main areas: economic growth efficiency, economic growth stability, economic structure optimization, and green development (Guo and Huang, 2020; Liu, 2021; Ren et al., 2021). Some scholars, however, have disagreed with these conclusions. They argue that industries sometimes agglomerate inefficiently and create problems such as insufficient resources and environmental carrying capacity, which have negative effects on GEE (Virkanen, 1998; Helsley and Strange, 2014; Huang, 2021). The third opinion type holds that the agglomeration impact on environmental pollution is diverse. Some studies have found that the impact of co-agglomeration on GEE has an “inverted U” curve (Wang and Sun, 2020; Ren et al., 2021), whereas others have found that the relationship between agglomeration and GEE was “U-shaped” (Yue et al., 2015; Zeng et al., 2021).

If the positive externality of co-agglomeration is greater than the negative externality, co-agglomeration behaves as promoting GEE, and vice versa, it behaves as inhibiting GEE. That is, the effect of co-agglomeration on GEE is related to the level of co-agglomeration, based on which, this study proposes the following hypothesis:

Hypothesis 1: The impact of corporate co-agglomeration on GEE is inverted U-shaped in China.

Co-agglomeration affects GEE through externalities, and one of the important externalities is that co-agglomeration promotes entrepreneurship, which influences economic growth and ecological environment by deploying social capital and assuming social responsibility (Omrane, 2015; Silvestri and Veltri, 2020).

Entrepreneurship is the sum of the entrepreneurs' abilities to identify potential opportunities, acquire resources, innovate, and implement actions that can drive enterprise development (Wennekers and Thurik, 1999). Agglomeration can promote entrepreneurship (Mason and Gos, 2014) because it provides entrepreneurs with the information needed to identify opportunities and establish social relationships (Audia and Rider, 2005; Kemper et al., 2011). Entrepreneurship enables companies to better deploy social capital, including trust, team effectiveness, and social relationships (Becchetti et al., 2022; Schlak, 2022). Entrepreneurial actions can improve mutual trust between members of the organization, the organizational actions become more efficient (Kacperska and Łukasiewicz, 2020; Sedrine et al., 2020). In addition, through their social network relationships, entrepreneurs can also overcome resource constraints and gain access to more resources and information (Bauernschuster et al., 2010), which leads to knowledge spillovers and collective learning between the agglomerated enterprises (Crespo et al., 2022), reduces the cost of technological innovation, and further improves productivity and boosts economic growth (Xu et al., 2021). Entrepreneurship also enables enterprises to take greater social responsibility (Chen et al., 2021; Biggeri et al., 2022). Entrepreneurs are the shapers of corporate culture and the leaders of corporate development (Mudrack, 2007). A great entrepreneur can lead by, for example, influence corporate culture, guide enterprises to achieve sustainable development, raise awareness of environmental protection, and contribute to economic transformation and social development (Branco and Rodrigues, 2006). A rising sense of social responsibility influences corporate decisions and disciplines company behavior, which in turn can motivate clean energy technology innovation, reduce pollution emissions, and protect the environment (Agudelo et al., 2020). Based on this logic, the second hypothesis is proposed to analyze the role of entrepreneurship in the impact process of co-agglomeration on GEE.

Hypothesis 2: Co-agglomeration can promote GEE through its spillover effects on entrepreneurship.

## Empirical Analysis

### Data Description

This paper selects 2000 to 2019 Chinese panel data^1^ as the research samples. The original data for the variables are extracted from the National Bureau of Statistics of China, China Statistical Yearbooks, China Labor Statistical Yearbooks, Provincial statistical Yearbooks, CEIC China Premium Database. To ensure the accuracy of the empirical results and weaken the dimensional differences, foreign direct investment (fdi), environmental regulations (er), and local fiscal expenditure on environmental protection (efe) are logarithmized.

### Variables Descriptions

#### Explained Variable: GEE

The GEE measures the overall output for both the economy and the environment and reflects green output environmental pollution costs. Specifically, the GEE measures the desirable output efficiency per unit of input costs and the environmental production process costs (Qian and Liu, 2013; Ohene-Asare and Turkson, 2019). This paper measures the GEE under constant scale conditions using the super-efficient SBM model (Tone, 2004; Lee, 2020), a method that appraises the relative efficiencies of multiple inputs and outputs (Kutty et al., 2022).

The measurement indicators are inputs, desirable outputs, and undesirable outputs, with the desirable outputs representing the desirable economic efficiencies, and the undesirable outputs representing the undesirable environmental efficiencies. The input factors are capital, labor, and energy inputs. The fixed asset investments, which are determined using the perpetual deposit method, are used to evaluate the capital stock and represent capital investment (Xie and Pan, 2011). The number of employees at the end of the year represents the labor, and the total energy consumption in each region is adopted to represent the energy input. The desirable output is the GDP in each region, and the undesirable output is the waste emissions. As most environmental pollution comes from the manufacturing sector, the undesirable outputs are represented by industrial wastewater, waste gas, and solid waste discharges, with the SO_2_ industry emissions being used to indicate the industrial waste gas discharges.

#### Explanatory Variable: Co-Agglomeration Index for the Corporations in Manufacturing and Producer Services^2^ Sector (Coaggl)

The co-agglomeration concept was proposed by Glaeser and Eillson. Based on an industrial geographic concentration measurement, Glaeser and Eillson constructed an E-G modified index to formulate a co-agglomeration index for two industries, which has consequently been widely used to assess co-agglomeration levels. In reference to the ideas proposed in Devereux et al. (2003) and Jiang and Xi (2014), this paper adopted the E-G correction index, which is calculated by incorporating the Herfindahl index and assigning weights, the formula for which is as follows:


$$\begin{array}{l} {\text{r}}_{\text{ij}}=\frac{{\text{H}}_{\text{ij}}-\left({\text{H}}_{\text{i}}\times {\text{w}}_{\text{i}}^{2}+{\text{H}}_{\text{j}}\times {\text{w}}_{\text{j}}^{2}\right)}{1-\left({\text{w}}_{\text{i}}^{2}+{\text{w}}_{\text{j}}^{2}\right)} \end{array}$$

where w_i_ and w_j_ are the weight index, which is the proportion of employees in a single industry to the sum of employees in two industries, H_i_, H_j_, and H_ij_, respectively, represent the geographic concentration formed by industry i, industry j, and two industries; the larger the value of r_ij_, the higher the agglomeration degree between the industries.

The geographic concentration is generally expressed using the Herfindahl index, for which the employment data from the various regions are adopted for the calculation, the formula for which is as follows:


$$\begin{array}{l} \text{HHI}=\overset{\text{n}}{\underset{\text{k}=1}{\sum }}{\text{S}}_{\text{k}}^{2}-\frac{1}{\text{n}} \end{array}$$

where S_k_ is the proportion of industrial employees in the entire region k, and n is the number of regions.

#### Control Variables

The first control variable is transportation infrastructure (tinfr) as convenient transportation can ease factor production constraints and increase productivity (Cedillo-Campos et al., 2022), for which the ratio of road kilometers to city area is used.

The second control variable is the industrial structure (is), which is measured based on the share of secondary sector output in total output. Generally speaking, the higher the secondary industry share, the more serious the pollution, and the greater the adverse effect on GEE (Muhammad et al., 2022).

The third control variable is the human capital level (hc). The greater the human capital improvement, the more efficient the knowledge dissemination, which is conducive to GEE improvements (Aljuboori et al., 2021; Martinidis et al., 2021). Therefore, the average education per capita is chosen to represent the labor quality in each region.

The fourth control variable is foreign direct investment (fdi) as this can affect regional innovative development and improve environmental quality (He, 2005; Ali et al., 2022) or can have a “pollution haven” effect [it refers to the tendency of companies in the industry to establish themselves in countries or regions where environmental standards are relatively low] (List and Co, 1999; Nguyen, 2021).

The fifth control variable is environmental regulations (er). Implemented government policies and regulations affect the environmental impacts on GEE. Moderate regulations can stimulate innovation and increase GEE (Lanoie et al., 2008; Thiel et al., 2016); however, excessive regulations can increase enterprise costs, which is not conducive to GEE improvement (Leiter and Winner, 2010; Saltari and Travaglini, 2011). Therefore, the proportion of corporate investment in pollution treatment to GDP is selected to represent the environmental regulations indicator.

The sixth control variable is the local fiscal expenditure on environmental protection (efe). The government can improve the environment by increasing its fiscal expenditure on corporations, which may lower corporate costs and motivate enterprises to improve their GEE.

### Research Design

To analyze the relationships between corporate co-agglomeration and GEE, a regression model with quadratic terms is established, as follows:


$$\begin{array}{l} \text{GE}{\text{E}}_{\text{it}}={\text{β}}_{0}+{\text{β}}_{1}\text{Coagg}{\text{l}}_{\text{it}}+{\text{β}}_{2}\text{Coagg}{\text{l}}_{\text{it}}^{2}+{\text{β}}_{3}\text{Contro}{\text{l}}_{\text{it}}+{\text{ε}}_{\text{it}} \end{array}$$

where GEE_it_ is the GEE in province i in year t, Coaggl_it_ is the co-agglomeration index in province i in year t, Coaggl${}_{\text{it}}^{2}$ is the quadratic term for the co-agglomeration index, Control_it_ represents the control variables, and ε_it_ is a random error term.

## Empirical Results

### Basic Regression

#### Descriptive Statistics

The descriptive data statistics for the regression analysis for the models in this paper are shown in Table 1.

**Table 1:** Descriptive statistics.

**Variable**	**Obs**	**Mean**	**Std. dev**.	**Min**	**Max**
GEE	600	0.8261264	0.2875783	0.2446396	1.262708
Coaggl	600	0.0529276	0.0116251	0.0144741	0.0955337
is	600	0.4287704	0.0804929	0.1598923	0.6196027
tinfr	600	0.0039737	0.0034247	0.0001219	0.0128906
hc	600	8.544943	1.091863	5.43834	12.782
fdi	600	55.61867	68.24681	0.0446	357.5956
er	600	0.0017699	0.0018004	7.45E-06	0.0185728
efe	600	77.86628	91.4546	0.06067	747.44
IE	600	7.688906	12.77725	0.1447019	88.22828
BE	600	0.0091846	0.0108836	0.000381	0.0813382

#### Main Results

This paper first uses the panel OLS method to determine the impact of the manufacturing and producer service co-agglomeration on GEE in China, the results for which are shown in Table 2. The Hausman test find that the Prob>chi^2^ for the four models are 0.0007, 0.0055, 0.0000, and 0.0000, respectively. As they all strongly reject the original hypothesis, the fixed effects model is chosen. The regression results in Table 2 show that the manufacturing and producer service co-agglomeration is conducive to the regional GEE improvements, and there is an “inverted U-shaped” relationship between the co-agglomeration and GEE. That is, the above empirical results indicate that hypothesis 1 of this paper is tested.

**Table 2:** Main results.

	**(1) GEE**	**(2) GEE**	**(3) GEE**	**(4) GEE**
Coaggl	4.113***	12.451***	5.046***	12.828***
	(2.968)	(3.022)	(3.601)	(3.210)
Coaggl^2^		−72.817***		−69.368***
		(−2.148)		(−2.078)
tinfr			46.145***	45.817***
			(5.013)	(4.991)
is			−0.347**	−0.383**
			(−1.684)	(−1.858)
hc			0.013	0.025
			(0.377)	(0.716)
logfdi			0.024***	0.024***
			(2.120)	(2.096)
er			8.563**	7.611**
			(1.862)	(1.652)
logefe			−0.038***	−0.037***
			(−2.905)	(−2.827)
_cons	0.755***	0.522***	0.513**	0.234
	(10.332)	(3.998)	(1.897)	(0.777)
Hausman test	14.44***	12.64***	44.47***	44.47***
P	0.0007	0.0055	0.0000	0.0000
Area fixed	Yes	Yes	Yes	Yes
Year fixed	Yes	Yes	Yes	Yes
N	600	600	600	600

t statistics in parentheses *p < 0.2, ***p < 0.05.

When the co-agglomeration level is within a proper range, competition and cooperation coexist, and agglomeration will generate externalities, such as increased cross-industry talent exchange, expanded knowledge, and technological spillover dissemination, and the innovative vitality of the enterprises is enhanced, thus leading to the enhancement of GEE (Chen and Hu, 2008; Liu and Rui, 2012). When the co-agglomeration level exceeds the range, its increase has an adverse impact on GEE because at this point, the market is overcrowded and threatens the carrying capacity of the region. The negative externalities outweigh the positive ones, and the economic aggregates are expressed as an increase in resource consumption and environmental pollution (Dou and Liu, 2016; Lin and Tan, 2019).

After adding the control variables, the co-agglomeration coefficient becomes larger, which indicates that the control variable selections are valid. Transportation infrastructure (tinfr) facilitates GEE as it can reduce circulation costs, optimize resource allocations, and promote talent and technology spillovers (Ghosh and Dinda, 2022). The secondary sector share depresses GEE because it generates greater pollution. Human capital (hc) can boost GEE, and industrial co-agglomeration provides conditions for the accumulation of human capital (Ji et al., 2021). As high-quality human capital represents knowledge, technology, and experience, it is more beneficial to knowledge and innovation spillovers (Yuan and Gao, 2020), which can elevate GEE. The foreign direct investment (fdi) coefficient is positive, which is inconsistent with the “pollution haven” hypothesis (Zeng and Zhao, 2009), and indicates that the benefits of foreign direct investment are greater than the drawbacks in China and can offer a better environment and conditions for regional GEE improvements. The environmental regulation coefficient is also positive, indicating that environmental regulations can have a positive adjustment effect on GEE, that is, environmental regulations regulate corporate behavior, reduce pollution emissions, and promote green technology innovation (Hamamoto, 2005; Telle and Larsson, 2006). The local government's fiscal expenditure on environmental protection significantly inhibits GEE improvements, which indicates that fiscal expenditure is an inefficient way to protect the environment as it weakens the incentive for enterprises to regulate themselves and improve the environment.

### Endogeneity Test

However, as there may have been an endogenous relationship between the co-agglomeration and the GEE, to deal with the endogeneity and ensure greater results reliability, this paper selects the instrumental variable method and redoes the estimation for which a lag of 1 is added to the co-agglomeration and a lag of 1 is added to the squared term as the instrumental variables. As shown in Table 3, the instrumental variables pass both the weak instrumental variable test and the non-identification test (Stock and Yogo, 2005); therefore, as the relationship between the co-agglomeration and GEE is still in an “inverted U-shape,” the instrumental variables are valid and the regression results are proven to be reliable.

**Table 3:** Instrumental variable regression.

	**Coaggl**	**Coaggl^**2**^**	**GEE**
Coaggl			11.243***
			(2.795)
Coaggl^2^			−60.043**
			(−1.811)
is			−0.327*
			(−1.558)
tinfr			45.521***
			(5.040)
hc			0.024
			(0.685)
logfdi			0.027***
			(2.289)
er			5.467
			(1.061)
logefe			−0.035***
			(−2.656)
IV1	1.011***		
	(12.78)		
IV2		0.8542***	
		(10.78)	
Kleibergen-Paap rk LM statistic			52.77
P			0
Cragg-Donald Wald F statistic			1,553.70
Kleibergen-Paaprk Wald F statistic			280.97
N			570

t statistics in parentheses *p < 0.2, **p < 0.1, ***p < 0.05.

### Heterogeneity Analysis

A heterogeneity analysis is conducted using group regression (Li and Song, 2008; Yang and Wang, 2014), the results for which are shown in Table 4. The suest test *p*-values show that the coefficients of variation between the regions are significant, indicating that there is heterogeneity between the regions^3^.

**Table 4:** Heterogeneity test.

**Heterogeneity** **test**		**Eastern** **region**	**Central** **region**	**Western** **region**	**Northeast** **region**
		**GEE**	**GEE**	**GEE**	**GEE**
Coaggl		14.441***	−39.265	104.766***	46.512*
		(3.086)	(−1.132)	(2.959)	(1.354)
Coaggl^2^		−124.183***	369.889	−862.152***	−242.827
		(−3.149)	(1.106)	(−2.889)	(−0.867)
tinfr		87.734	42.150	51.020***	−17.178
		(0.950)	(1.096)	(4.649)	(−0.755)
is		−2.763***	1.487***	0.811*	0.875***
		(−5.574)	(2.558)	(1.408)	(4.463)
hc		0.154***	−0.012	−0.015	0.011
		(2.569)	(−0.128)	(−0.262)	(0.156)
logfdi		0.123***	−0.059*	0.003	−0.004
		(3.190)	(−1.396)	(0.167)	(−0.258)
er		−5.389	−27.441*	12.992*	4.003
		(−0.779)	(−1.388)	(1.642)	(0.592)
logefe		0.095***	−0.280***	−0.047*	−0.003
		(2.429)	(−2.637)	(−1.359)	(−0.118)
_cons		−1.721***	1.236*	−2.825***	−1.190
		(−2.884)	(1.109)	(−2.408)	(−0.954)
N		200	100	210	60
Area fixed		Yes	Yes	Yes	Yes
Year fixed		Yes	Yes	Yes	Yes
Suest test *P*-value	Eastern region	1	–	–	–
	Central region	0.1417*	1	–	–
	Western region	0.0044***	0.0028***	1	–
	Northeastern region	0.1127*	0.0389***	0.1185*	1

t statistics in parentheses *p < 0.2, ***p < 0.05.

As shown in Table 4, the influence of co-agglomeration on GEE in the eastern, western, and northeastern regions has an “inverted U-shaped” curve. The difference is that the influence of co-agglomeration on GEE in the central region has a “U-shaped” curve, which is because in the central region, when the co-agglomeration is at a lower level, the negative externality generated by agglomeration is greater than the positive externality, resulting in lower GEE. However, as the level of co-agglomeration continues to rise, the GEE will increase.

Currently, individual provinces in the eastern region have had excessively high co-agglomeration in the recent years, which has inhibited the GEE. The corporate co-agglomeration in most other provinces and cities is still promoting GEE. The industrial co-agglomeration in the central regions is at the bottom of the “U-shaped” curve and indicates that the current co-agglomeration does not adequately contribute to the GEE. The industrial co-agglomeration in the western region has crossed to the left side of the “inverted U-shaped” curve, which is not conducive to GEE improvements. In the northeastern region, the co-agglomeration level is at the top of the “inverted U” curve, which will inhibit GEE if it continues to grow.

In addition, the co-agglomeration in the central, western, and northeastern regions has a greater impact on GEE than in the eastern region. In other words, for every 1% point change in the co-agglomeration index, the degree of change in the GEE in the central, western, and northeastern regions is greater than in the eastern region. This is because the eastern region has a better economic and industrial structure than the central, western, and northeastern regions, which weakens the marginal promotion effect of co-agglomeration on GEE. However, the central, western, and northeastern regions have a larger marginal effect because of the larger optimization space.

### Robust Test

To verify the robustness of the above results, the sample is changed and another method is adopted to re-estimate the model. First, system GMM is chosen as the new estimation method because system GMM can deal with heteroscedasticity problems and weaken endogeneity. Second, to lessen the effects of the sample time span and extreme data, the 2019 sample was excluded^4^ and the explanatory variables are cut by 1% up and down. As shown in Table 5: (1) the conclusion that industry co-agglomeration promotes regional GEE is still proven; (2) the “inverted U-shaped” curve is still significant; and (3) the coefficients for the other variables remain unchanged. Therefore, the above conclusions are proven to be robust.

**Table 5:** Robust test.

	**(1) GMM method**	**(2) Changing the samples**
	**GEE**	**GEE**
Coaggl	14.490***	16.627***
	(2.984)	(3.216)
Coaggl^2^	−83.606**	−102.687***
	(−1.882)	(−2.145)
is	−0.325**	−0.281*
	(−1.705)	(−1.433)
tinfr	45.349***	46.896***
	(6.375)	(6.180)
hc	0.027	0.018
	(0.836)	(0.551)
logfdi	0.027***	0.020*
	(2.178)	(1.590)
er	5.463	4.285
	(0.874)	(0.689)
logefe	−0.035***	−0.032***
	(−2.776)	(−2.542)
_cons	0.175	0.174
	(0.494)	(0.489)
AR	1	–
Hansen test P	0.980	–
Area fixed	Yes	Yes
Year fixed	Yes	Yes
*N*	570	540

t statistics in parentheses *p < 0.2, **p < 0.1, ***p < 0.05.

### Mechanism Test

To further investigate whether entrepreneurship is the transmission mechanism for co-agglomeration on GEE, the mediating effect model proposed by Baron and Kenny (1986) is consulted to test. Considering the importance of the many connotations of entrepreneurship and the availability of data, this paper draws on the approach of Hébert and Link (1989) to classify entrepreneurship into business entrepreneurship (BE) and innovation entrepreneurship (IE).

The total number of manufacturing and producer service patents granted in each region is divided by the annual population of the region to obtain the number of granted patents per 10,000 people, which is then used to represent the innovation entrepreneurship (IE) (Wong et al., 2005; Li et al., 2009; Song and Chen, 2020). The number of private firms in manufacturing and producer services per 10,000 people is taken to represent business entrepreneurship (BE) (Beugelsdijk and Noorderhaven, 2004; Glaeser, 2007; Ovaska and Takashima, 2021).

Referring to the mediating effects test model (Wen et al., 2004), Table 6 is generated, which shows the results of the mechanism test for innovation entrepreneurship and business entrepreneurship.

**Table 6:** Mechanism test.

	**(1) GEE**	**(2) IE**	**(3) GEE**	**(4) BE**	**(5) GEE**
Coaggl	5.474***	61.499*	5.321***	0.234***	4.543***
	(3.871)	(1.342)	(3.766)	(6.558)	(3.123)
tinfr	46.145***	488.032*	45.370***	0.762***	44.508***
	(5.013)	(1.672)	(4.917)	(3.251)	(4.791)
is	−0.347*	−46.514***	−0.273	−0.040***	−0.262
	(−1.684)	(−7.121)	(−1.267)	(−7.550)	(−1.209)
hc	0.021	7.578***	0.002	0.005***	0.003
	(0.584)	(6.644)	(0.047)	(5.146)	(0.093)
logfdi	0.016*	−0.302	0.016*	0.000	0.024**
	(1.347)	(−0.805)	(1.414)	(0.733)	(2.080)
er	9.958***	329.616***	9.138**	0.197*	8.140*
	(2.121)	(2.166)	(1.943)	(1.681)	(1.766)
logefe	−0.050***	−1.067***	−0.047***	0.000	−0.039***
	(−4.143)	(−2.738)	(−3.905)	(1.273)	(−2.972)
IE/BE			0.00249**		2.774**
			(1.888)		(1.73)
_cons	0.495*	−56.716***	0.636**	−0.030***	0.577**
	(1.919)	(−6.788)	(2.374)	(−4.312)	(2.099)
N	600	600	600	600	600

t statistics in parentheses *p < 0.2, **p < 0.1, ***p < 0.05.

In Table 6, it can be seen that the test results of columns (2) and (4) are significantly positive, indicating that the positive contribution of co-agglomeration to entrepreneurship is significant. The test results of columns (3) and (5) are also significantly positive, indicating that the mediating effect of entrepreneurship is significant. Among them, the mediating effect of IE is 2.8% and the mediating effect of BE is 11.9%. That is, co-agglomeration can spill over to entrepreneurship, and entrepreneurship promotes GEE. Entrepreneurship, as one of the externalities of co-agglomeration, optimizes the efficiency of resource allocation within enterprises and strengthens knowledge spillover and technological innovation. Meanwhile, excellent entrepreneurs guide enterprises to pursue efficient and sustainable development by enhancing their sense of responsibility and mission. Therefore, co-agglomeration can promote GEE through its spillover effects on entrepreneurship, and hypothesis 2 of this paper is tested.

## Conclusion

Using 2000–2019 panel data from China, this paper measures GEE and corporate co-agglomeration level and empirically investigates the effects of co-agglomeration on GEE, from which the following are discovered. First, the manufacturing and producer service corporate co-agglomeration is significantly conducive to enhancing GEE in China, with the corporate co-agglomeration showing an “inverted U-shaped” relationship with the GEE. Second, there is regional heterogeneity in the effects of corporate co-agglomeration on GEE. In the central region, the corporate co-agglomeration does not contribute sufficiently to the GEE. The co-agglomeration in the western and northeastern regions and some developed eastern provinces is found to be too high. Finally, entrepreneurship plays a mediating role in the effect of corporate co-agglomeration on GEE.

The findings in this paper indicate that the level of co-agglomeration needs to be controlled to better promote GEE and entrepreneurship is an important factor in this process. Therefore, local policies should be carefully designed to have a guiding effect on corporate location decisions. Green industrial parks and tax incentive policies should be established to support the co-agglomeration of corporates. Meanwhile, by taking the carrying capacity of local infrastructure and resources into consideration, focusing on the quality of FDI, and exerting the constraining effect of environmental regulations, the local government could avoid the negative effects of excessive co-agglomeration. In addition, institutions and policies that facilitate the exercise of entrepreneurship should be formulated. Practical measures such as improving the financial system, providing funding support, and lowering the threshold for business startups could be taken to encourage the exertion of entrepreneurship.

## Data Availability Statement

The datasets presented in this study can be found in online repositories. The names of the repository/repositories and accession number(s) can be found in the article/supplementary material.

## Author Contributions

XZ, YZ, and WY contributed to the conception and design of the study. YZ organized the database and performed the statistical analysis. All authors wrote the first draft of the manuscript together, contributed to manuscript revision, read, and approved the submitted version.

## Conflict of Interest

The authors declare that the research was conducted in the absence of any commercial or financial relationships that could be construed as a potential conflict of interest.

## Publisher's Note

All claims expressed in this article are solely those of the authors and do not necessarily represent those of their affiliated organizations, or those of the publisher, the editors and the reviewers. Any product that may be evaluated in this article, or claim that may be made by its manufacturer, is not guaranteed or endorsed by the publisher.

## Footnotes

^1^Tibet is not included because of the lack of data. Taiwan, Hong Kong, and Macao are excluded because their statistical systems differ from the mainland's.

^2^There is no specific statistical data for producer services. We refer to the Statistical Classification of producer services (2019) and the categories of Statistical Yearbook, and select transportation, warehousing and postal services, information transmission, software and information technology services, financial services, leasing and business services, scientific research and technical services, and real estate to represent producer services sector.

^3^Eastern region, central region, western region and northeastern region in the Yearbook are divided as following: Eastern 10 provinces (municipalities) include: Beijing, Tianjin, Hebei, Shanghai, Jiangsu, Zhejiang, Fujian, Shandong, Guangdong and Hainan; Central 6 provinces include: Shanxi, Anhui, Jiangxi, Henan, Hubei and Hunan; Western 12 provinces (autonomous regions and municipalities) include: Inner Mongolia, Guangxi, Chongqing, Sichuan, Guizhou, Yunnan, Tibet(Not included in this article due to missing data), Shaanxi, Gansu, Qinghai, Ningxia and Xinjiang; Northeastern 3 provinces include: Liaoning, Jilin and Heilongjiang.

^4^In 2019, the National Development and Reform Commission issued a directory of industrial restructuring, guiding the focus on promoting a new development concept and promoting the high-quality transformation of the manufacturing industry; therefore, these data were removed for the robustness to reduce the impact of the sample time span.
